# An Open-Label, Pilot Trial of HRG80™ Red Ginseng in Chronic Fatigue Syndrome, Fibromyalgia, and Post-Viral Fatigue

**DOI:** 10.3390/ph15010043

**Published:** 2021-12-29

**Authors:** Jacob Teitelbaum, Sarah Goudie

**Affiliations:** Kona Research Center, 76-6322 Kaheiau St., Kailua Kona, HI 96740, USA; appointments@endfatigue.com

**Keywords:** fatigue, fibromyalgia, chronic fatigue syndrome, post-viral CFS, Asian ginseng, pain, pain relief

## Abstract

Chronic fatigue syndrome and fibromyalgia (CFS/FMS) affect 2.1% of the world’s population and ~10–25% of people who have had COVID-19. Previous clinical data suggested that a unique *Panax ginseng* (C.A. Meyer, family Araliaceae) root extract (HRG80™ Red Ginseng) often resulted in marked improvement. We aimed to study this hydroponic form of red ginseng root, containing high levels of rare ginsenosides, for improving energy, cognition, and stamina. This open-label prospective study included participants with severe CFS/FMS who took a daily supplement of HRG80 capsules (200–400 mg) or tablets (100–200 mg) for one month. A total of 188 subject patients completed the one-month treatment trial. Of these, 60.1% rated themselves as improved, with 13.3% rating themselves as being much better. In this group, the mean composite score improved from 11.9 to 18.8 (*p* < 0.001), with a 67% average increase in energy, 44% average increase in overall well-being, 48% average improvement in mental clarity, 58% average composite improvement in the previous three measurements (primary outcome measure), 46% average improvement in sleep, 33% average decrease in pain, and 72% average increase in stamina. Our study showed that HRG80 red ginseng root powder resulted in a marked improvement in people with CFS and fibromyalgia. This included the subgroup with post-viral CFS/FMS.

## 1. Introduction

Several factors have combined to stress human energy production, including but not limited to:

1. A major loss of micronutrients in the processed Western diet, including as much as an 85% loss of the key energy nutrient magnesium [1].
2. Insufficient sleep [2].
3. The increased speed and stress of modern life.
4. Infections triggering chronic fatigue syndrome and fibromyalgia (CFS/FMS).

Historically, in Asian medicine, Asian ginseng root has been one of the most valued herbs for overall support of energy and vitality. This has been discussed at length in reviews elsewhere [3,4].

Oral ginsenosides are poorly absorbed [5]. Traditionally, high doses (e.g., 3000–9000 mg/day) of ginseng have been recommended [6]. This may sometimes limit ginseng’s clinical utility. Therefore, methods of increasing the absorption of these therapeutic agents may be helpful. One clinically studied system is the use of the natural plant compound gamma cyclodextrin [7], which has been combined with HRG80 Red Ginseng in chewable tablets. This was 1 of the 2 formulations used in this study. The study includes separate evaluations of the subject’s self-reported responses to each of these two formulations.

The literature suggests that these benefits may be amplified by using a new hydroponically grown ginseng (HRG80), which the producing company’s research suggests has as much as seven times higher levels of rare ginsenosides than other ginseng products [8].

Rare ginsenosides are suggested to have higher bioavailability and biological activity than classic ginsenosides [8]. This led the authors to explore the use of HRG80 Red Ginseng in their clinical practice, where many patients treated withHRG80 Red Ginseng reported it to be extremely helpful. Therefore, we decided to conduct an initial pilot study to assess its efficacy in CFS and fibromyalgia, including separately exploring its effects in a subset of the study group with non-COVID-19 triggered post-viral CFS/FMS. More information on the levels of ginsenosides in HRG80, as well as its production, can be found elsewhere [8].

Because of the high ginsenoside levels in HRG80, as well as the availability of a chewable tablet with gamma cyclodextrin to enhance absorption of the ginseng, we wanted to further explore what forms and dosing offered the optimal clinical benefit. We also wanted to explore whether lower doses could be used to produce beneficial effects similar to those seen using much higher doses of other ginseng preparations.

In addition to the 33% of adults who already complain of having major fatigue [9], there has been a dramatic increase in both CFS and fibromyalgia associated with COVID-19 [10]. CFS/FMS are also worsened by the total ongoing stress load (both physical and emotional) on the body. This “allostatic load” is increased in both CFS [11] and fibromyalgia [12]. An allostatic load is viewed as a reflection of the effects on the system of chronic increases in cortisol [13].

Red ginseng has shown increased effectiveness for fatigue and cognition relative to other forms of ginseng [8,14]. It may be especially helpful for stress-induced psychological fatigue as well [15]. Importantly, this may be via modulation of the cortisol stress response via the hypothalamic–pituitary–adrenal (HPA) axis [16], which is significantly impaired in CFS [17], showing a decreased adrenal gland response to CRH stimulation. In vitro, HRG80 also exhibited positive effects on neuroinflammation, senescence, apoptosis, and immune responses, which were found even in a wide range of low concentrations [18].

The Sars-CoV-2 virus responsible for the COVID-19 illness has accentuated allostatic load. It has achieved this by a number of mechanisms, including increased emotional stress. Since the onset of the virus, surveys have shown that over 40% of the population has some degree of anxiety, and 41.3% has depression [19]. In addition to this increase in symptoms of emotional stress, it is estimated that 10 to 20% of people with symptomatic COVID-19 will have persistent post-viral CFS [10] following their infection. The Director of NIAID, Dr. Anthony Fauci, has noted that these persistent post-COVID-19 symptoms are highly suggestive of post-viral CFS [20]. Research by the study author (J.T.) showed that post-viral CFS/FMS responds similarly to CFS/FMS caused by other triggers [21].

This dramatic increase in post-viral fatigue associated with COVID-19 makes the need for effective support urgent. Key hallmarks of CFS/FMS, including post-COVID-19 and post-viral chronic fatigue, include severe fatigue and cognitive dysfunction (sometimes called “brain fog”) [22]. Both may benefit from intervention with *Panax ginseng* (C.A. Meyer, family Araliaceae) root extracts [4,8,14,15]. This study explores whether HRG80 Red Ginseng can help those with CFS/FMS (including post-viral forms).

## 2. Results

A total of 188 participants completed the treatment. All continuous variables were normally distributed, and no missing data were observed. The sample characteristics are summarized in Table 1. Briefly, the sample was, on average, 59 years old with a mean duration of illness of 19 years. Most subjects were female and most met criteria for chronic fatigue syndrome and fibromyalgia. More than half of the patients included in the sample were characterized as improvers.

For the primary and secondary analyses, there was a significant mean increase in Visual Analog Scale (VAS) composites and all VAS subscale scores from pre- to post-treatment, with predominately large effect sizes (see Table 2). The same pattern of findings was observed in the subsample of improvers, though all the effect sizes were notably larger (see Table 3). No post-study follow-up was conducted. The results of the supplementary analyses examining changes in VAS scores via the virus onset of the illness did not reveal any significant group-by-treatment interactions (all *p* values > 0.05). Likewise, there were no significant group-by-treatment interactions when examining change in VAS scores by supplement form (capsule vs. tablet, all *p* values > 0.05). These findings can be taken to mean that treatment effects were similar across groups. The results of the mixed model ANOVAs are summarized in Table 4 and Table 5.

Exploratory analyses examining treatment effects by varying supplement doses and forms did not reveal any significant group-by-treatment interactions in the overall sample (all *p* values > 0.05). There was no statistically significant difference among any of the four subgroups (i.e., 1 capsule, 1 tablet, 2 capsules, and 2 tablets per day) in terms of the degree of change on the VAS scores (composite or subscales). This can be taken to mean that 100 mg (in the form of 1 tablet daily) was very clinically effective and statistically significant, and there was no added benefit to taking more than 100 mg per day of red ginseng when it was combined with the gamma-cyclodextrin (present only in the tablet form, not capsules). This is further supported by comparisons between those who took 1 tablet and 1 capsule per day in the subgroup of improvers, which did not reveal any significant differences, indicating that most of the highly significant improvement can be obtained by taking a single tablet of 100 mg of the HRG80 Red Ginseng combined with the gamma-cyclodextrin daily (see Table 6; additional supporting data are available on request).

Descriptive summary statistics are provided for self-reported daily supplement preferences in Table 7. To briefly summarize, just over half of the sample reported taking 2 capsules or tablets per day, while 70% of the sample indicated that they preferred to take the supplements once daily. There was a significant overall difference in preference for daily doses between those who used capsules and those who used tablets (X2 = 7.78, *p* = 0.020). Specifically, a greater proportion of persons preferred two supplements per day (compared to one) when administered in capsule form, as compared to tablet form. There was no difference between groups in their preference for the frequency of daily dose.

**Figure 1:**
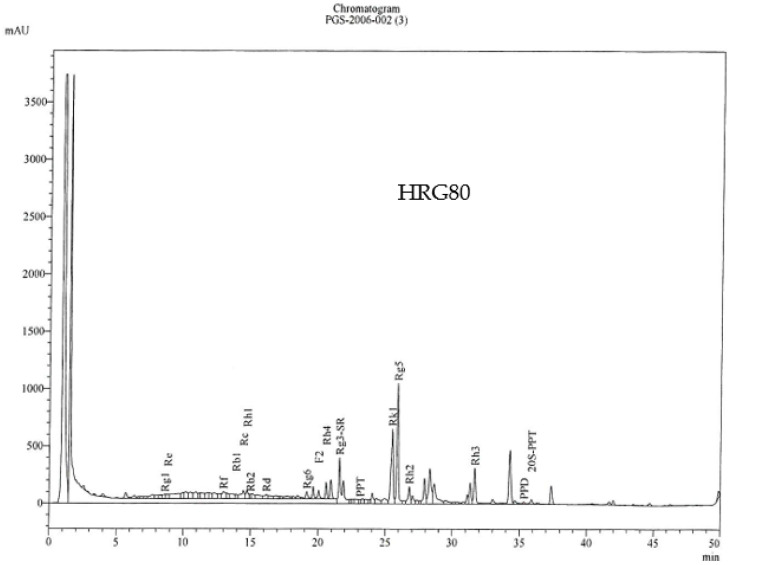
HRG80^TM^ HPLC fingerprint and composition (Item 11 in Supplementary Materials of [18]).

Consistency of total and rare ginsenosides between batches (Table 8) is acceptable. Table 9 and Figure 1 show the HPLC fingerprint and composition of the HRG80.

### 2.1. Generalizability

The study population reflected a diverse population of people in the United States suffering with CFS and fibromyalgia. So, we expect it to be generalizable to the US population.

### 2.2. Dropouts and Side Effects

The study received 258 applications to participate from people who met the inclusion criteria. The enrollment instructions noted that they could stop treatment at any time; however, if they did, they were asked to provide us their reasons why.

Six participants notified us of their decision to drop out of the study. Two said the supplement was overstimulating. One experienced nausea and headaches and was concerned that it might be raising their blood pressure (although they did not check their blood pressure). A fourth subject reported feeling better on the supplement but discontinued taking it after experiencing some chest heaviness, having had a history of mitral valve prolapse. One never took it after learning the tablet contained xylitol. Another stopped because they found that their blood sugar dropped to 61 mg/dl and was concerned that it was causing hypoglycemia.

Overall, 19 people noted side effects that did not clear by simply adjusting the dose. Seven found it overstimulating. Three had diarrhea. One had constipation. Three had worsening of their headaches. One person noted sleepiness, spaciness, and acne. Two had PMS or irritability. One noted a drop in their blood sugar to a blood sugar of 61 mg/dl. One had pain and fatigue that worsened.

Despite repeated requests for follow-up information, 64 participants did not fill out the post-study questionnaire or offer any information as to why they did not. Their data was, therefore, not included in the study results.

## 3. Discussion

This study provides preliminary data on a potentially effective herbal intervention for chronic fatigue syndrome and fibromyalgia, including patients who suffer from these conditions following viral infections. It is very promising that this intervention was able to significantly improve patients’ clinical outcomes, with 60.1% of subjects improving from their pre-treatment status.

The group that improved reported an average 67% increase in energy, 72% increase in stamina, and 48% improvement in mental clarity, which is a substantial improvement as the result of taking a single agent. This makes HRG80 Red Ginseng a powerful addition to our S.H.I.N.E.^®^ Protocol, which optimizes Sleep, Hormonal function, Immunity, Nutritional support, and limited Exercise, as patients are able. In our earlier randomized placebo-controlled study, the S.H.I.N.E.^®^ protocol resulted in an average of 91% improvement in quality-of-life of those with CFS/FMS [21,23], making these conditions highly treatable.

Research has now advanced to the point where fatigue, brain fog, and other health conditions associated with CFS and fibromyalgia can be significantly improved.

This study has several weaknesses, a key one being the lack of randomization and a placebo control group. Another is that outcomes were all subjective. In addition, of the 258 people who initially qualified for the study, ~25% of these (64) were lost during the follow-up, despite repeated attempts to contact them. It is possible that they did not offer post-study data because of observed side effects or a lack of response to the intervention.

About 18% of studies have ‘lost to follow-up’ rates of over 20% [24]. It would not be unexpected for this to occur more frequently in community-based studies with low amounts of personal contact with the subjects, and where there is no financial payment given to the subjects for completing the study.

In addition to supporting the effectiveness of the specific ginseng preparation, the study also adds important information about the use of the HRG80 form of the herb and explores the kind of dosing that works best for this form of ginseng. A significant problem in the use of ginseng is the relatively high doses (often 3000–9000 mg) sometimes required to achieve optimal benefits [6].

Clinically, we found much lower doses of HRG80 to be effective, typically in the range of 100–400 mg. This was supported by a recent study [8] showing that the effective total daily dose of HRG80 Red Ginseng in treating day-to-day fatigue was 418 mg.

Marked clinical and statistical improvement was reported by all groups taking the HRG80 ginseng. However, when combined with gamma-cyclodextrin (GammaSorb™) to enhance absorption, most of the statistically significant clinical improvement was obtained with a single tablet containing just 100 mg of the red ginseng root powder. Increasing the HRG80 Red Ginseng dose from 100 mg to as high as 400 mg (without the gamma-cyclodextrin) resulted in clinically modest but statistically insignificant improvements relative to the 100 milligram tablets. However, the form and dose found to be optimal varied with each individual, as is typical in clinical practice. Overall, this study shows that even doses as low as 100 mg of HRG80 Red Ginseng can deliver optimal benefits when combined with gamma cyclodextrin.

## 4. Materials and Methods

### 4.1. Patient Enrollment

This paper’s lead author, Jacob Teitelbaum MD, has a clinical practice that specializes in treating CFS/FMS. His newsletters (available at Vitality101.com accessed on 17 November 2021), which also focuses on these conditions, is available worldwide to people with these conditions. Recruitment using the newsletter was felt to allow a very broad base, increasing the generalizability of the data. Subjects needed to have severe illness, rating their overall wellbeing as 5/10 or less on their VAS, generally signifying an over 50% decrease in function. The author (J.T.) invited his patients as well as readers of his newsletter to participate in this study. Those taking non-ginseng treatments were instructed to continue these without any changes throughout the study period. A total of 188 participants qualified for the study by meeting the inclusion criteria below, being willing to take the supplement, completing the online pre-study questionnaire, and agreeing to complete the post-study questionnaires. Subjects filled out and submitted the online pre-and post-questionnaires remotely with no assistance from the study authors or their team. These questionnaires were designed using Google Forms. No compensation was offered or given for participating in the study, aside from the fact that participants received the supplement free of charge.

### 4.2. Inclusion Criteria

1. Subjects needed to meet the American College of Rheumatology (ACR) 2010 (amended 2011) diagnostic criteria for fibromyalgia [25] or the CDC (Centers for Disease Control and Prevention) criteria for chronic fatigue syndrome [26].
2. Subjects needed to rate their overall well-being as 5 or less on the Visual Analogue Scale (VAS).
3. Those with post-viral fatigue were also allowed to participate.
4. Subjects needed to live in the United States, be over 18 years of age, and nonpregnant.

### 4.3. Exclusion Criteria

1. Subjects could not be taking the blood thinner Coumadin (generic name: warfarin).
2. Subjects could not be pregnant.

### 4.4. Objectives and Randomization

**Figure 2:**
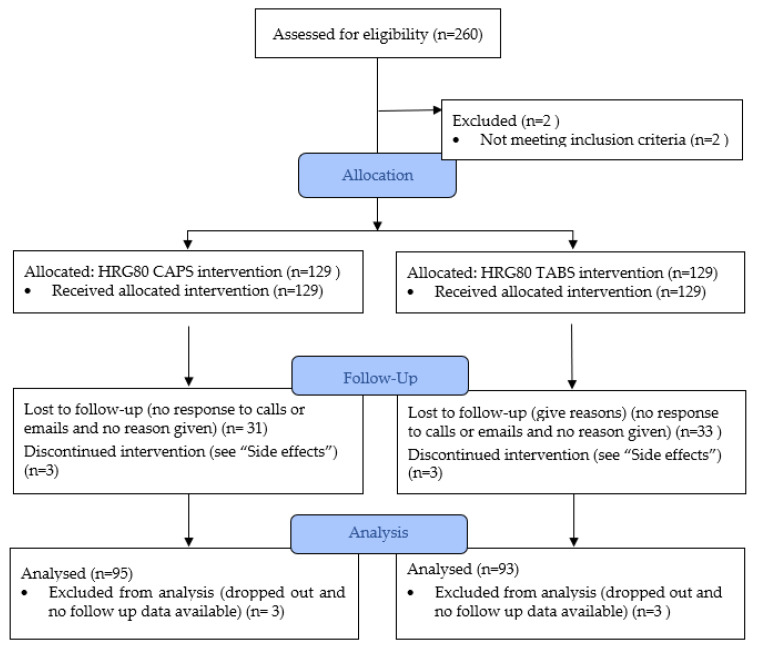
Flow Diagram.

No placebo group was used as this was an open trial which aimed, predominantly, to compare the subject’s CFS/FMS symptoms pre- and post-treatment. It also sought to specifically determine if the effect was different in the post-viral CFS/FMS subgroup. It was hypothesized that, since red ginseng was shown to help energy and cognition in earlier studies [4,8,14,15], it might be helpful for patients with CFS/FMS. A secondary objective was to compare the effectiveness of two forms of the herb (tablets versus capsules) and clinically determine the dosing that the subjects felt to be optimal. Randomization into the capsule and tablet groups (see Figure 2) was conducted using the simple expedient of having the shipping department, who had no clinical information whatsoever about the subjects and no contact with the study authors, randomly ship tablets to half of the subjects and capsules to the other half. Allocation concealment was achieved by recording which subjects received tablets versus those who received capsules in a different state from the authors’ location.

### 4.5. Sample Size

The sample size of study participants was set based on the constraints of how many bottles of red ginseng were available for the study.

### 4.6. Interventions

Participants were given HRG80 Red Ginseng for one month of use, beginning between 15 January and 8 March 2021. Subjects were sent either Terry Naturally^®^ HRG80 Red Ginseng Energy capsules (manufactured by EuroPharma, Inc. under the brand name Terry Naturally^®^), which contain 200 mg Panax ginseng root powder (HRG80) per capsule (without GammaSorb™) or Terry Naturally HRG80 Red Ginseng Energy chewable tablets, which contain 100 mg of the HRG80 Red Ginseng root powder. The 100 mg chewable tablets also contained gamma-cyclodextrin (GammaSorb™) to enhance absorption. One of the purposes of the study was to compare the effectiveness of this ginseng both with and without the GammaSorb™.

Subjects were instructed to take either one or two tablets or capsules daily. If taking two daily, they could be taken together, or subjects could take one in the morning and one at lunch time. They were instructed to adjust the dosing to what they found to be clinically optimal, without specifying a timetable or further instructions for doing so.

Subjects were instructed to continue any prescription or supplement treatments that they were taking at the beginning of the study, but to not add any new ones during the month of treatment.

### 4.7. Outcome Measures

The primary outcome measure was the VAS composite score measured pre- and post-treatment from a sum of three subscales listed below (energy, well-being, and mental clarity). Secondary outcome measures were the individual changes in energy, well-being, mental clarity, sleep, pain, and stamina. The subjects also indicated their overall self-assessment of being much worse, worse, no change, better, or much better.

The VAS questions asked were as follows:

(A) How is your energy?
  1 2 3 4 5 6 7 8 9 10
  1 = “near dead” and 10 = excellent
(B) How is your overall sense of well-being?
  1 2 3 4 5 6 7 8 9 10
  1 = “near dead” and 10 = excellent
(C) How is your mental clarity?
  1 2 3 4 5 6 7 8 9 10
  1 = “brain dead” and 10 = good clarity
(D) How is your sleep?
  1 2 3 4 5 6 7 8 9 10
  1 = no sleep and 10 = 8 h of sleep a night without waking
(E) How severe is your achiness/pain (1 is worst possible pain)?
  1 2 3 4 5 6 7 8 9 10
  1 = very severe pain and 10 = pain free
(F) How is your stamina?
  1 = no stamina and 10 = healthy stamina

### 4.8. Study Design

The design (a prospective, open, unblinded trial) used outcome measures in the form of the VAS questionnaire and was kept simple to improve compliance. All patients gave informed consent, and the study was approved by the Practitioner Alliance Network IRB (ID#: PAN-HRG80–2020; 1 August 2020).

Patients could continue their current treatments during the study. They were asked not to make any changes to their current treatments during the study.

### 4.9. Statistical Analysis

All analyses were performed using the Statistical Package for the Social Science (SPSS) version 27.0 (SPSS Inc., IBM Corp., Armonk, NY, USA). Continuous variables were assessed for normality using a visual inspection of histograms and qq plots. The primary outcome measure was the VAS composite score measured pre- and post-treatment. The composite score was derived from a sum of three subscales (energy, well-being, and mental clarity), ranging from 0 to 30, with higher scores indicating better health and functioning. Secondary outcomes were measured from pre- and post-treatment total scores on six VAS subscales (energy, sleep, pain, well-being, mental clarity, and stamina), ranging from 0 to 10, with higher scores indicating better health and functioning. Two-tailed, paired-sample *t*-tests were used to examine subjects’ changes in VAS scores following treatment with the alpha level set to 0.05. Effect sizes were calculated using Cohen’s d for paired samples t-tests. Effect sizes are interpreted as follows: small: d = 0.2; medium: d = 0.5; and large: d = 0.8. To adjust for multiple testing in relation to the secondary outcome analyses, a Bonferroni Correction was applied with the alpha level set to 0.008 (0.05/6). Analyses were performed for the overall sample (*n* = 188) and repeated in the subsample that self-reported feeling “better” or “much better” following treatment (herein referred to as improvers; *n* = 113).

Supplementary analyses were conducted to examine whether changes in VAS scores were different between subjects whose illness onset was, in the subject’s opinion and experience, accompanied by what they perceived to be severe viral symptoms. A mixed model ANOVA (analysis of variance) was conducted, with treatment entered as a within-subjects factor (i.e., pre- and post-treatment VAS scores) and illness onset status (yes = 76, no = 112) entered as a between-subjects factor. The group-by-treatment interaction was examined to determine whether the effect of treatment was different between the illness onset groups. A second mixed model ANOVA was conducted to examine the differences in treatment effects between subjects who took the supplements in capsule (*n* = 95) versus tablet (*n* = 93) form. The mode of supplement was entered as the between-subjects factor, and the group-by-treatment interaction was examined. The assumption of equality of variances was assessed with Levene’s test.

Additionally, exploratory analyses were conducted to further understand the effects of different supplement forms and doses. A mixed model ANOVA was run with treatment as the within-subjects factor and dose/form as a four-group between-subjects factor (1 tablet per day = 35; 1 capsule per day = 22; 2 tablets per day = 39; and 2 capsules per day = 59). Follow-up analyses were conducted among the subsample of improvers to describe and compare the effectiveness of treatment among specific subgroups using *t*-tests. A series of chi-square tests was used to examine the differences in daily preference for supplement intake. Specifically, the frequencies of preferred daily dose (one per day, two per day, other) and daily frequency (once daily, twice daily, other) were compared between subgroups who used capsules or tablet form. The alpha level was set to 0.05 for all supplementary and exploratory analyses.

## 5. Conclusions

HRG80 Red Ginseng resulted in marked improvements in energy levels, sleep, mental clarity, pain relief, and overall well-being in those with CFS and fibromyalgia, including those with post-viral CFS/FMS. These findings need to be replicated in a double-blind placebo-controlled study. 

## Figures and Tables

**Table 1:** Sample Characteristics (*n* = 188).

Variable	Value
Age in years, mean (SD)	58.6 (11.6)
Gender, female %	89.4 (168)
Duration of illness in years, mean (SD)	18.9 (10.6)
Onset with viral illness, % (n)	40.4 (76)
Diagnosis of CFS, % (n)	96.8 (182)
Diagnosis of FMS, % (n)	94.1 (177)
Self-reported feeling post-treatment, % (n)	
Much worse Worse Same Better Much better	2.7 (5) 3.2 (6) 34.0 (64) 46.8 (88) 13.3 (25)

Note: CFS: chronic fatigue syndrome; FMS: fibromyalgia.

**Table 2:** Change in VAS Scores with Treatment in Full Sample (*n* = 188).

Variable	Pre-tx Mean (SD)	Post-tx Mean (SD)	t Statistic (*p*-Value)	Effect Size (*d*)	Percent Improvement
VAS Composite *	12.2 (3.5)	17.1 (4.8)	−14.31 (<0.001)	−1.04	40.2
VAS 1. Energy	3.6 (1.3)	5.4 (1.8)	−13.30 (<0.001)	−0.97	50.0
VAS 2. Sleep	4.5 (1.7)	6.0 (2.0)	−10.46 (<0.001)	−0.76	33.3
VAS 3. Pain	4.6 (1.8)	5.5 (1.8)	−5.84 (<0.001)	−0.43	19.6
VAS 4. Well-Being	3.9 (1.4)	5.7 (1.9)	−12.89 (<0.001)	−0.94	46.2
VAS 5. Mental Clarity	4.7 (1.6)	6.1 (1.8)	−10.18 (<0.001)	−0.74	29.8
VAS 6. Stamina	3.3 (1.5)	4.8 (2.1)	−10.04 (<0.001)	−0.73	45.5

Note: VAS: Visual Analogue Scale. * Composite is sum of Energy, Well-Being, and Mental Clarity.

**Table 3:** Change in VAS Scores Among Subsample Who Self-Reported Improvement (*n* = 113).

Variable	Pre-tx Mean (SD)	Post-tx Mean (SD)	t Statistic (*p*-Value)	Effect Size (*d*)	Percent Improvement
VAS Composite *	11.9 (3.2)	18.8 (4.3)	−17.71 (<0.001)	−1.67	58.0
VAS 1. Energy	3.6 (1.4)	6.0 (1.7)	−14.86 (<0.001)	−1.40	66.7
VAS 2. Sleep	4.4 (1.7)	6.4 (1.8)	−11.95 (<0.001)	−1.36	45.5
VAS 3. Pain	4.5 (1.9)	6.0 (1.7)	−7.36 (<0.001)	−0.90	33.3
VAS 4. Well-Being	3.8 (1.2)	6.3 (1.6)	−15.63 (<0.001)	−1.74	43.6
VAS 5. Mental Clarity	4.4 (1.3)	6.5 (1.7)	−12.80 (<0.001)	−1.45	47.7
VAS 6. Stamina	3.2 (1.4)	5.5 (2.0)	−12.48 (<0.001)	−1.41	71.9

Note: VAS: Visual Analogue Scale. * Composite is sum of Energy, Well-being, and Mental Clarity.

**Table 4:** Results of Mixed Model ANOVAs Examining Change in Primary and Secondary Outcomes by Illness Onset Status (N = 188).

Outcome Variable	F-Statistic (*p*-Value)
VAS Composite	
Treatment Treatment * Group	204.57 (<0.001) 1.57 (0.212)
VAS 1. Energy	
Treatment Treatment * Group	172.22 (<0.001) 0.26 (0.614)
VAS 2. Sleep	
Treatment Treatment * Group	113.03 (<0.001) 2.77 (0.098)
VAS 3. Pain	
Treatment Treatment * Group	31.47 (<0.001) 0.33 (0.567)
VAS 4. Well-Being	
Treatment Treatment * Group	168.03 (<0.001) 2.03 (0.156)
VAS 5. Mental Clarity	
Treatment Treatment * Group	104.74 (<0.001) 1.49 (0.224)
VAS 6. Stamina	
Treatment Treatment * Group	103.65 (<0.001) 2.37 (0.125)

Note: VAS: Visual Analogue Scale. Group: Onset with viral illness—yes (*n* = 76), no (*n* = 112). The “*” notation reflects “treatment times group.” This interaction term (treatment X group) provides an estimate of whether the effect of treatment in one group is statistically different from the treatment effect in another group.

**Table 5:** Results of Mixed Model ANOVAs Examining Change in Primary and Secondary Outcomes by Supplement Form (N = 188).

Outcome Variable	F-Statistic (*p*-Value)
VAS Composite	
Treatment Treatment * Group	203.65 (<0.001) 0.04 (0.844)
VAS 1. Energy	
Treatment Treatment * Group	220.65 (<0.001) 2.44 (0.120)
VAS 2. Sleep	
Treatment Treatment * Group	109.70 (<0.001) 1.19 (0.277)
VAS 3. Pain	
Treatment Treatment * Group	33.91 (<0.001) 0.58 (0.447)
VAS 4. Well-Being	
Treatment Treatment * Group	165.39 (<0.001) 0.02 (0.891)
VAS 5. Mental Clarity	
Treatment Treatment * Group	103.00 (<0.001) 0.18 (0.676)
VAS 6. Stamina	
Treatment Treatment * Group	100.85 (<0.001) 1.82 (0.179)

Note: VAS: Visual Analogue Scale. Group: Supplement form was capsules (*n* = 95), tablet (*n* = 93). The “*” notation reflects “treatment times group”.

**Table 6:** Percentage Improvement in Each Symptom (in Improvers) with Each Dose and Form.

Outcome Variable (% Improvement in Improvers)	One Tablet (*n* = 25)	One Capsule (*n* = 16)	Tablet vs. Capsule t Statistic (*p*-Value)	Effect Size (*d*)	Two Tablets (*n* = 24)	Two Capsules (*n* = 39)
VAS Composite *	60.6	69.0	0.56 (0.581)	0.18	70.7	68.3
VAS 1. Energy	70.9	78.4	0.31 (0.757)	0.11	92.1	96.5
VAS 2. Sleep	62.5	78.8	0.64 (0.531)	0.23	59.4	60.9
VAS 3. Pain	45.5	67.0	0.90 (0.375)	0.29	36.2	67.2
VAS 4. Well-Being	68.5	80.6	0.61 (0.547)	0.20	79.6	80.1
VAS 5. Mental Clarity	56.4	67.9	0.70 (0.490)	0.22	55.0	58.7
VAS 6. Stamina	74.7	123.7	1.41 (0.168)	0.45	87.8	105.1

Note: VAS: Visual Analogue Scale. * Composite is sum of Energy, Well-Being, and Mental Clarity.

**Table 7:** Daily Supplement Intake Preferences.

	Full Sample (*n* = 188)	Subgroup: Capsule (*n* = 95)	Subgroup: Tablet (*n* = 93)
Daily dose, % (n)			
One per day Two per day Other	30.3 (57) 52.1 (98) 17.6 (33)	23.2 (22) 62.1 (59) 14.7 (14)	37.6 (35) 41.9 (39) 20.4 (19)
Daily frequency, % (n)			
Once per day Twice per day Other	70.2 (132) 17.6 (33) 12.2 (23)	74.7 (71) 16.8 (16) 8.4 (8)	65.6 (61) 18.3 (17) 16.1 (15)

**Table 8:** HRG80 Red Ginseng: Consistency between Batches. Three random recent batch reports were picked to compare the amounts of total ginsenosides and rare ginsenosides between batches of the HRG80 ginseng. Results are as follows.

Batch	Total Ginsenosides (%) *	Rare Ginsenosides (%) **
7 September 2021	10.6%	10.1%
9 July 2021	12.6%	11.7%
26 May 2021	12.5%	10.6%

* Total ginsenosides: Rb1, Rb2, Rb3, Rc, Rd, Re, Rf, Rg1, Rg2, Rg3, Rg5, Rg6, Rh1, Rh2, Rh3, Rh4, Rk1, Rk2, Rk3, Ro, CK, PPD, PPT, F1, F2, and F4; typical value: 12 %. ** Rare ginsenosides: Rg2, Rg3, Rg5, Rg6, Rh1, Rh2, Rh3, Rh4, Rk1, Rk2, Rk3, CK, PPD. PPT, F1, F2; Typical value 10%.

**Table 9:** HRG80 Red Ginseng: The composition (%) of powdered herbal preparations.

Ginsenosides	Content (%) in HRG80 ^TM,^
Rg1	0.023
Re	0.114
Rf	0.081
Rb1	0.046
Rg2	0.00
Rc	0.473
Rh1	0.071
Rb2	0.130
F1	0.005
Rd	0.477
Rg6	0.218
F2	0.131
Rh4	1.092
Rg3-(S-R)	1.080
PPT (20-R)	0.022
Rk1	1.009
C(k)	0.00
Rg5	1.888
Rh2	0.232
Rh3	0.455
20S-PPT	0.015
PPD	0.038
**Total**	**7.599**

## Data Availability

The data presented in this study are available on request from the corresponding author. The data are not publicly available due to HIPAA and privacy regulations.
